# Isolation and characterization of bacteriophages from soil against food spoilage and foodborne pathogenic bacteria

**DOI:** 10.1038/s41598-023-36591-6

**Published:** 2023-06-07

**Authors:** Putri Christy Artawinata, Sesilia Lorraine, Diana Elizabeth Waturangi

**Affiliations:** 1 Food Technology Department, Faculty of Biotechnology, Atma Jaya Catholic University of Indonesia, Jenderal Sudirman 51 Street, South Jakarta, DKI Jakarta 12930 Indonesia; 2 Master in Biotechnology Department, Faculty of Biotechnology, Atma Jaya Catholic University of Indonesia, Jenderal Sudirman 51 Street, South Jakarta, DKI Jakarta 12930 Indonesia

**Keywords:** Biotechnology, Microbiology, Molecular biology

## Abstract

Microbial food spoilage and foodborne disease are the main challenges in the food industry regarding food shelf life. Current preservation methods are frequently associated with changes in organoleptic characteristics and loss of nutrients. For this reason, bacteriophage offers an alternative natural method as a biocontrol agent that can reduce bacterial contamination in food without altering the organoleptic properties. This study was conducted to isolate and characterize bacteriophage from soil to control food spoilage bacteria, such as *Bacillus cereus* and *Bacillus subtilis*, and foodborne pathogenic bacteria, such as enterotoxigenic *Escherichia coli* (ETEC) and enterohemorrhagic *E. coli* (EHEC). Isolation was done by agar overlay assay method, and phages BC-S1, BS-S2, ETEC-S3, and EHEC-S4 were recovered. The host range of all isolated phages tended to be narrow and had high specificity towards the specific bacteria. The phage efficiency were measured where ETEC-S3 showed no effectivity against *B. cereus* and EHEC-S4 showed low efficiency against Enteropathogenic *E. coli* (EPEC). Morphology analysis was conducted for phage BC-S1 and ETEC-S3 with Transmission Electron Microscopy (TEM), and it is shown to belong to the *Caudovirales* order. Phages BC-S1 and BS-S2 significantly reduced the host bacteria when applied to the cooked rice and pasteurized milk samples with miMOI of 0.1. While phage ETEC-S3 at miMOI of 0.001 and phage EHEC-S4 at miMOI of 1 also showed a significant reduction when applied to chicken meat and lettuce samples at storage temperatures of 4 °C and 28 °C. The highest bacterial reduction of 100% was shown by phage BC-S1 on pasteurized milk samples and reduction up to 96.06% by phage ETEC-S3 on chicken meat samples at 28 °C incubation.

## Introduction

Microbial food contamination is a major concern in the food industry. Contaminated food could contain a variety of microbes, including bacteria that can use food as an energy source, causing neither food spoilage nor foodborne disease^1^. Food spoilage can result in any changes in the sensory characteristics of a product that causes food to be undesirable for consumption. A wide variety of metabolic by-products that cause off-odor, off-flavor, also color, and textural changes may lead to food loss, causing considerable economic and environmental effects^2^. *Bacillus* sp. groups, such as *Bacillus cereus* and *Bacillus subtilis* are spore-forming bacteria whose spores can survive the high processing temperature. Commonly found in many spoiled foods, such as ropiness in bread, slime formation in rice, also off-odor in milk^3,4^. Although spoiled foods may be safe to eat, some bacteria could have pathogenicity that leads to foodborne diseases. One of the main pathogens involved in diarrheal disease, which can also lead to death, is Diarrheagenic *Escherichia coli* (DEC) which can be found in soil and water, such as ETEC and EHEC^5^.

Many risk factors associated with bacterial food contamination are often related to its processing, preparation, storage, and handling practices. Conventional food preservation methods, such as pasteurization, high-pressure processing, irradiation, and chemical or biological agents, are commonly used to help improve food safety. However, those treatments are frequently associated with changes in organoleptic characteristics, loss of nutrients, also toxic-threatening side effects^6,7^. Additionally, even with the variety of methods available, foodborne outbreaks still occur relatively often^8^. For this reason, finding an alternative preservation method to control food spoilage is required.

One promising and safe technique that addresses several shortcomings is bacteriophage biocontrol. This method uses lytic bacteriophages to specifically target pathogenic bacteria and eliminate or significantly reduce their levels in food in order to enhance the safety of food products. Bacteriophages are viruses that lyse living bacterial hosts. This lytic potential has been exploited in attempts to design a more natural antimicrobial approach to control bacteria at the various stages of food production^9^. They are highly host-specific, safe to consume, relatively inexpensive, and do not alter the organoleptic properties of food^10^. They can be found almost everywhere where live bacteria exist, such as soil, offering the possibility to isolate them for therapeutic purposes. Hence, the use of bacteriophages as alternative natural preservation is very promising^6,11^. Based on these backgrounds, this study aimed to isolate and characterize bacteriophages from the soil in controlling food spoilage and foodborne pathogenic bacteria, such as *B. cereus, B. subtilis,* ETEC, and EHEC.

## Results

### Bacteriophage isolation from soil and titer determination

*Bacillus cereus* phage S1 (BC-S1), *Bacillus subtilis* phage S2 (BS-S2), ETEC phage S3 (ETEC-S3), and EHEC phage S4 (EHEC-S4) were isolated from different soil samples near organic waste disposal. The clear plaques formed as the result of the agar overlay assay indicated the lysis of bacteria by phage. The isolated bacteriophages concentration were measured through titer determination. Phage BC-S1 performed highest titer with the value of 1.72 ± 0.31 × 10^10^ PFU/mL compare with phage BS-S2 1.57 ± 0.92 × 10^9^ PFU/mL, phage ETEC-S3 8.24 ± 1.38 × 10^9^ PFU/mL, and also with phage EHEC-S4 with the value of 1.26 ± 0.86 × 10^5^ PFU/mL.

### Host range determination

The isolated bacteriophages host range were determined using *B. cereus*, *B. subtilis*, ETEC, EHEC, EPEC, and *Vibrio cholerae*. The host range of isolated bacteriophages was showed in Table 1. Besides their capability to lyse its host cell, phage ETEC-S3 also showed lytic activity against *B. cereus* and phage EHEC-S4 showed lytic activity against EPEC. While phage BC-S1 and phage BS-S2 showed that they could only lysis their host itself. They performed high host specificity that could not attack other bacteria, even of which belonged to the same genus.

**Table 1:** Bacteriophages host range.

Bacteriophage	Spectrum host cell bacteria					
	BC	BS	ETEC	EHEC	EPEC	VC
BC-S1	+	−	−	−	−	−
BS-S2	−	+	−	−	−	−
ETEC-S3	+	−	+	−	−	−
EHEC-S4	−	−	−	+	+	−

S1, soil 1; S2, soil 2; S3 , soil 3; S4, soil 4.

### Efficiency of plating (EOP)

All isolated phages showed activity only in infecting specific bacteria, their bacterial host. However, phage ETEC-S3 was also found to be inefficiently attack *B. cereus* with EOP lower than 0.001, while phage EHEC-S4 also performed low efficiency with EOP 0.001–0.2 against EPEC (Table 2).

**Table 2:** Bacteriophages efficiency of plating (EOP).

Bacteriophage	Target bacteria					
	BC	BS	ETEC	EHEC	EPEC	VC
BC-S1	1.0	−	−	−	−	−
BS-S2	–	1.0	−	−	−	−
ETEC-S3	0.000001 ± 0.000005	−	1.0	−	−	−
EHEC-S4	−	–	–	1.0	0.11 ± 0.10	–

Data were shown in mean ± standard error value.

### Minimum inhibitory multiplicity of infection (miMOI)

**Figure 1:**
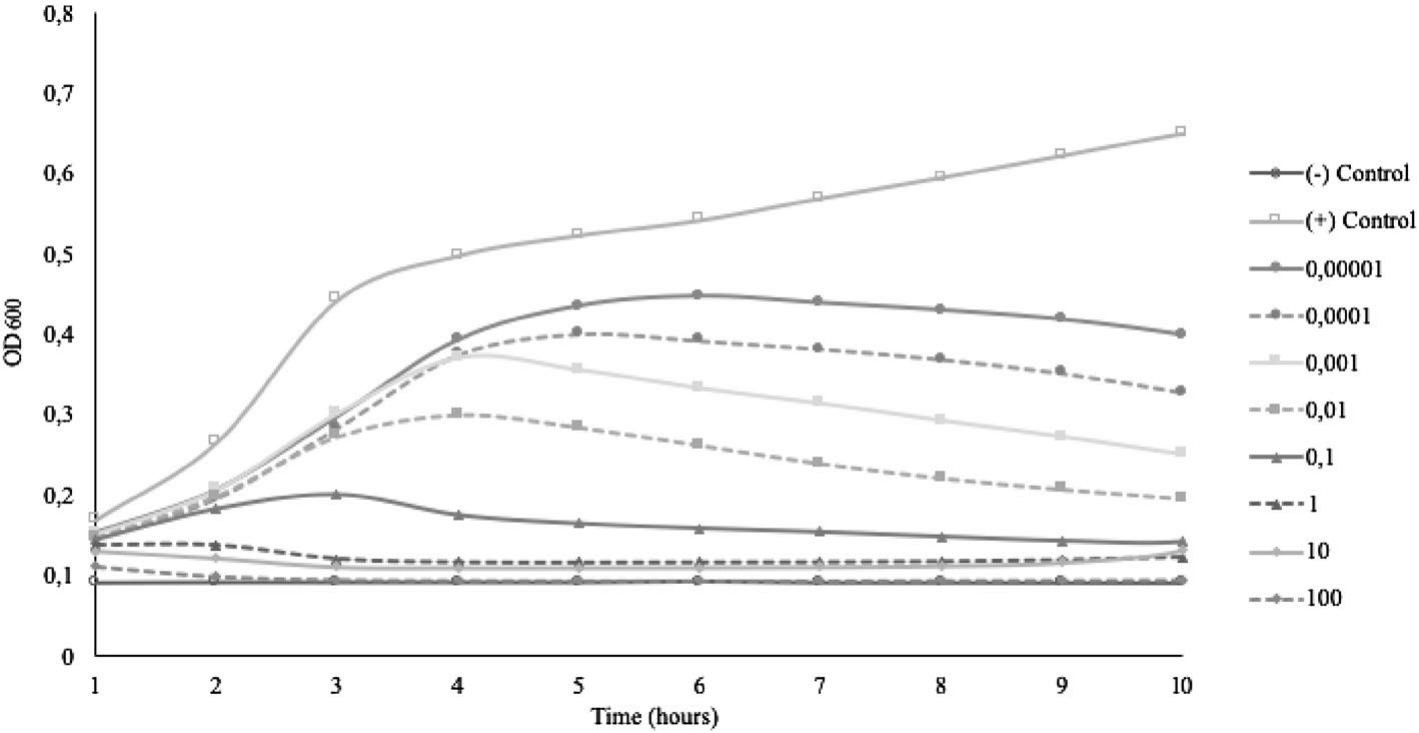
miMOI of Bacteriophage BC-S1.

**Figure 2:**
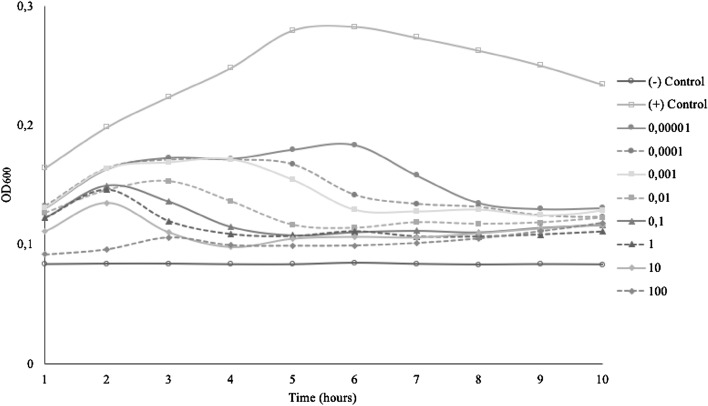
miMOI of Bacteriophage BS-S2.

**Figure 3:**
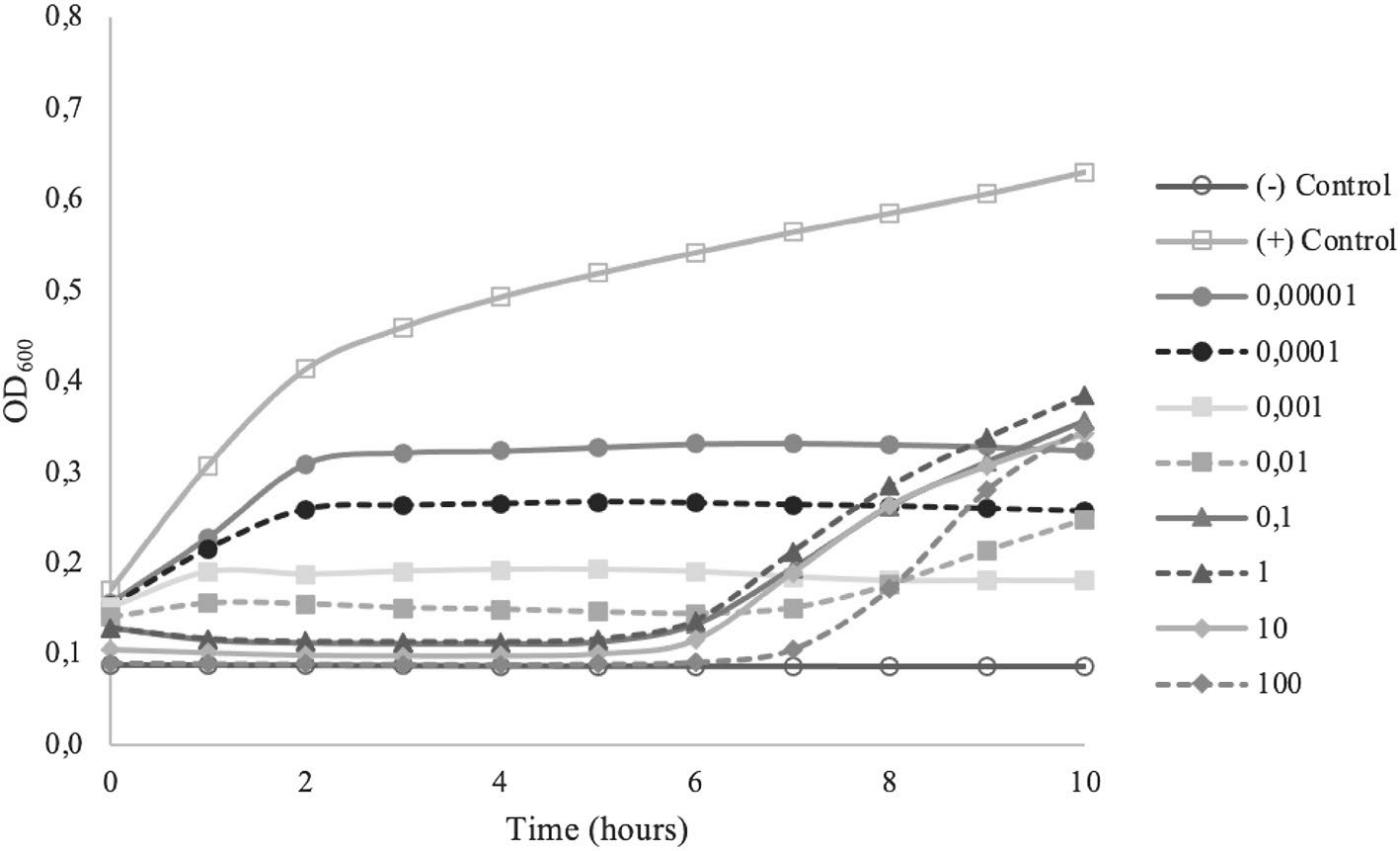
miMOI of Bacteriophage ETEC-S3.

**Figure 4:**
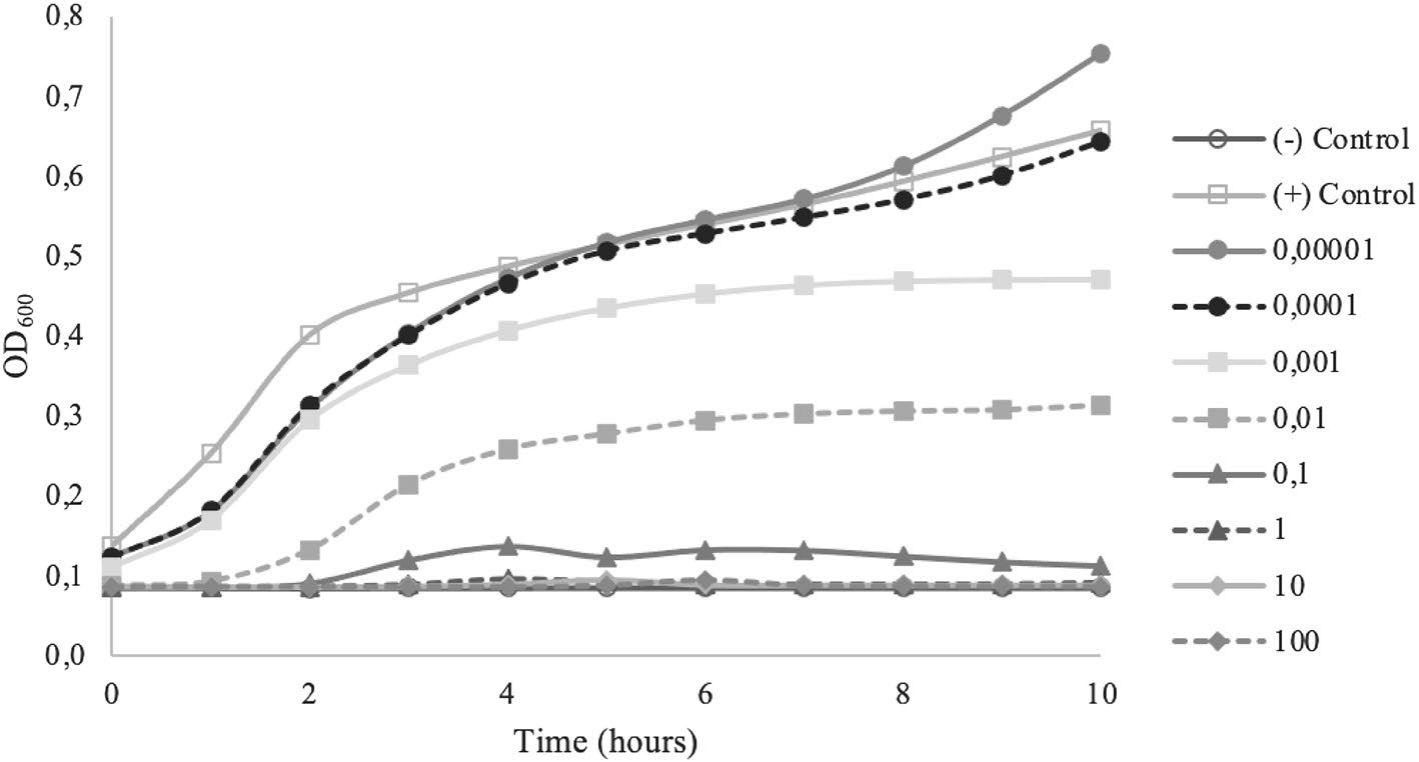
miMOI of Bacteriophage EHEC-S4.

The bacteriophage MOI was carried out on eight different concentrations from 10^2^ to 10^–5^. Positive control showed only host bacteria without adding bacteriophages, while negative control showed only bacteriophages without adding the host bacteria. Based on the result, the highest inhibition for phage BC-S1 (Fig. 1) and phage BS-S2 (Fig. 2) were shown for MOI 0.1. While ETEC-S3, MOI 0.01 to the highest MOI 100 can inhibit ETEC with no growth in the first 6 h of incubation, followed by re-growth of ETEC. Therefore, miMOI of phage ETEC-S3 was 0.001, whereas the graphical showed no bacterial growth of ETEC (Fig. 3). miMOI of phage EHEC-S4 was 1, which could inhibit the growth of EHEC within 10 h of incubation. Bacterial growth decreased as the MOI increased (Fig. 4).

### Morphology analysis

**Figure 5:**
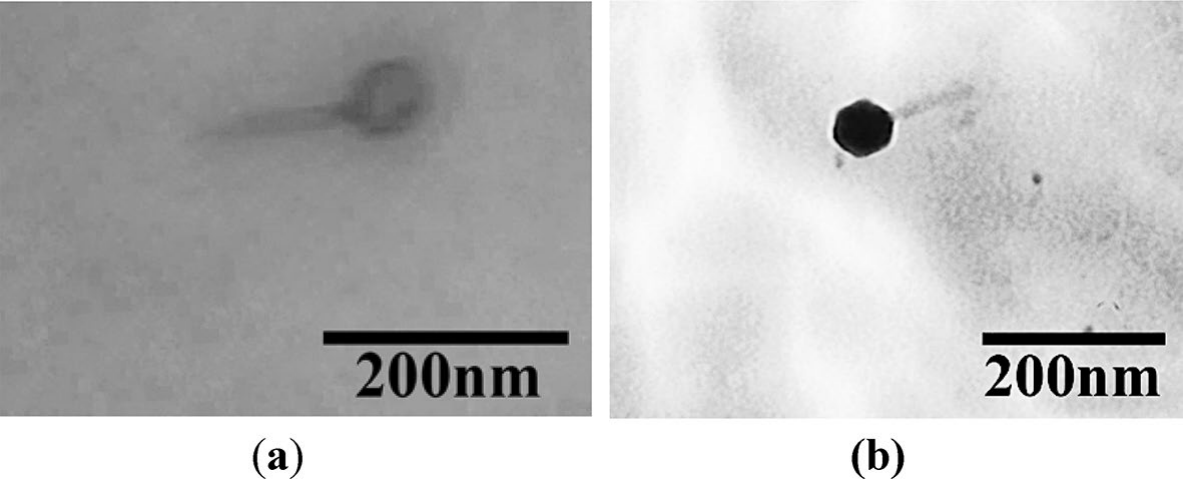
Bacteriophages morphology with TEM (**a**, BC-S1; **b**, ETEC-S3).

Bacteriophage BC-S1 and phage ETEC-S3 were continued for morphology determination due to their higher activities using transmission Electron Microscopy (TEM) as shown in Fig. 5. Phage BC-S1 performed an icosahedral head with about 75 nm diameter and about 90 nm contractile tail. While phage ETEC-S3 performed an icosahedral head with about 65 nm diameter and about 100 nm contractile tail.

### Bacteriophage application on food samples

Each bacteriophage was tested for its ability to reduce the number of specific pathogenic bacteria on food samples. Phages BC-S1 and BS-S2 were applied to cooked rice and pasteurized milk, while Phages ETEC-S3 and EHEC-S4 were applied to chicken meat and lettuce to see the effect of their application on different food matrices and surfaces. The incubation was carried out overnight at refrigerated storage temperature (4 °C) and room temperature (28 °C). The results were shown in Table 3, where all isolated bacteriophages showed a reduction in all samples and treatments. The highest reduction percentage by phages BC-S1 and BS-S2 was found on pasteurized milk samples, while phages ETEC-S3 and EHEC-S4 showed the highest reduction on chicken meat samples at 28 °C of storage temperature.

**Table 3:** Bacteriophage application onto a variety of food samples with different storage temperatures.

Phage	Samples	Temperature (°C)	Control (CFU/mL)	Treatment (CFU/mL)	Bacterial reduction (log)	Bacterial reduction (%)
BC-S1	Cooked rice	4	3.48 ± 0.01 × 10^5a^	2.27 ± 0.05 × 10^5a^*	0.19	34.74
		28	2.67 ± 0.17 × 10^8c^	0.97 ± 0.06 × 10^8b^*	0.44	63.85
	Pasteurized milk	4	6.91 ± 0.75 × 10^5a^	2.36 ± 0.11 × 10^5a^*	0.46	65.47
		28	3.89 ± 0.01 × 10^7b^	4.54 ± 0.24 × 10^4a^*	2.93	99.88
BS-S2	Cooked rice	4	1.02 ± 0.04 × 10^5d^	0.76 ± 0.08 × 10^5d^*	0.14	26.24
		28	9.54 ± 0.01 × 10^7e^	0.81 ± 0.09 × 10^7e^*	1.08	91.47
	Pasteurized milk	4	2.48 ± 0.65 × 10^5d^	1.65 ± 0.59 × 10^5d^*	0.19	32.30
		28	8.85 ± 0.12 × 10^7e^	0.75 ± 0.03 × 10^7e^*	1.07	91.53
ETEC-S3	Chicken Meat	4	1.01 ± 0.09 × 10^7a^	5.89 ± 1.49 × 10^6a^*	0.23	41.41
		28	4.85 ± 0.11 × 10^9d^	1.91 ± 0.05 × 10^8e^*	1.40	96.06
	Lettuce	4	8.82 ± 0.15 × 10^6a^	6.15 ± 0.20 × 10^6a^*	0.16	30.29
		28	2.91 ± 0.05 × 10^8b^	4.67 ± 0.16 × 10^7c^*	0.79	83.93
EHEC-S4	Chicken Meat	4	2.08 ± 0.03 × 10^4a^	1.37 ± 0.01 × 10^4a^*	0.18	34.25
		28	1.27 ± 0.09 × 10^9c^	1.51 ± 0.02 × 10^8d^*	0.93	88.14
	Lettuce	4	3.74 ± 0.09 × 10^5a^	1.82 ± 0.08 × 10^5a^*	0.31	51.51
		28	1.80 ± 0.10 × 10^8ab^	3.06 ± 0.11 × 10^7b^*	0.77	82.98

Data were shown in mean ± standard error value.

Different letters in each column indicated significant differences, α ≤ 0.05.

“*”Indicate significant differences between control and bacteriophage treatment for each sample.

## Discussions

Bacteriophages are the most abundant life form on earth and can be found in nearly every habitat, such as soil, water, and food. Soil near organic waste disposal was used as a sample because it is an ideal source to isolate bacteriophages since it contains high numbers of diverse bacteria. Thus, the presence of its bacteriophages is potential. There are an estimated 1.5 × 10^8^ bacteriophages per gram of agricultural soil^12^.

Four bacteriophages were successfully recovered, namely phage BC-S1 against *B. cereus*, phage BS-S2 against *B. subtilis*, phage ETEC-S3 against ETEC, and phage EHEC-S4 against EHEC. These phages could be considered lytic bacteriophages due to their clear plaque formation in the agar overlay assay. However, in areas where bacteriophages are absent, the bacteria grow to the stationary phase and form a confluent, opaque layer or “lawn” in the soft agar overlay^13^. Some required criteria for applying bacteriophage as a biocontrol agent are obligately lytic, non-transducing, and toxin gene-free to ensure safety^14^. Lytic bacteriophages replicate by attaching, injecting nucleic acid, and lysing the host cell to produce the phage progeny. The “newborn” bacteriophages are then ready to start another cycle by infecting another bacteria cell. While in the lysogenic cycle, phages integrate their nucleic acid into the chromosome of the host cell and replicate with it as a unit without destroying the cell^15^. Therefore, bacteriophages should be obligately lytic to reduce the potency of toxin gene transfer that can increase their virulence^16^.

Knowing the concentration of the isolated bacteriophages is essential. Bacteriophage titer is one of the factors that can affect their effectiveness in phage therapy applications. A high titer value indicates better phage stability. Bacteriophage applications for therapeutic use require a high titer of lytic phages (10^9^ PFU/mL)^17^. In this study, the titer of isolated bacteriophages varied from 10^5^ to 10^10^ PFU/mL. It is recommended to refresh the bacteriophage regularly to maintain the phage stability in a high titer because long-time storage may cause the titer to decline^18^.

Characterization of bacteriophage by host range determination is also one factor in evaluating the isolated phage ability against different strains of host bacteria^11^. Phage ETEC-S3 was found to have the capability to infect *B. cereus*, and phage EHEC-S4 can infect EPEC, but both showed low efficiency. All the isolated bacteriophages performed a highly specific or narrow host range, including phage BC-S1 or BS-S2, since it showed high lytic activity only for the bacterial host, while to several pathogenic bacteria showed low efficacy and showed no lytic activity on the others, even more. Generally, newly isolated bacteriophages can only infect hosts with the same general receptor type as the isolated host^14^. The location of the cell receptors varies depending on the phage and the host. It can be located on the cell wall, flagella, pili, capsules, or surface protein in bacteria cells. Phage cannot bind to the host cell if the receptors are inaccessible or non-complementary to the phage receptor binding protein^19^. Other studies suggest that nearly all bacteriophages isolated using a single host strain of bacteria may be more likely to have a narrow rather than broad host range. In many cases, this narrow property is desirable because it usually has great specificity to the host itself, preventing the killing of other species of bacteria and leaving the rest of the bacterial host intact. This narrow host range phage is also essential for phage cocktail development, which broadens the host range for phage therapy. In this case, characterization of the individual phage of the cocktail is needed^14,20^.

The Efficiency of Plating (EOP) was used to define the effectiveness of bacteriophage against target bacteria. EOP value of 0.5–1.0 is ranked as high efficiency, an EOP value of 0.2 to 0.5 is categorized as medium efficiency, while an EOP value of 0.001 to 0.2 is classified as low efficiency, and EOP below 0.001 is inefficient^21^. According to the result, phage EHEC-S4 performed higher efficiency than phage ETEC-S3. Phage ETEC-S3 was considered ineffective towards *B. cereus*, while phage EHEC-S4 performed low efficiency towards EPEC. Both isolated phages showed high specificity since they showed high activity only against bacterial hosts. Low EOP can be caused by the action of host resistance systems blocking the intracellular virus development or due to poor bacteriophage adsorption to the host cells^22^.

MOI is determined as PFU/CFU ratio, which counts only adsorbed phages attached to and then infected bacteria^23^. The minimum MOI value needs to be examined to determine the potential effective concentration that can be used. In this study, all isolated phages showed different optimum MOI to inhibit or lysis each host completely. The minimum MOI value obtained for phage BC-S1 and BS-S2 were 0.1, the MOI of phage ETEC-S3 was 0.001, and the MOI of phage EHEC-S4 was 1. Phage ETEC-S3 showed the lowest MOI compared with the others, indicating that phage ETEC-S3 is considered more effective because a lower phage concentration is needed to reduce the number of bacteria. Effective MOI value is affected by environmental conditions such as the number of infecting phages, the number of target cells to attach, also how fast and how much time is allowed for attachment^12,23^. Phage BC-S1, BS-S2, and EHEC-S4 gave a better result in lytic activity using higher MOI numbers. Bacterial growth decreased as the MOI increased because higher use of MOI enhances the probability of phage particles infecting their host bacteria. A lower MOI value still showed the reduction though it could not completely inhibit the bacterial host growth. More concentration of bacteriophages means more cells can be lysed, yielding rapid lytic activity^24^. However, these phages differed from phage ETEC-S3, where MOI higher than 0.001 could not completely inhibit the growth of the cells. The bacterial reduction was shown in the first 6 h of incubation, and then the bacteria continued to re-grow. Phage concentration that is too high in terms of MOI may lead to bacterial lysis via the enzymatic action of the phage lysins once the phages are attached to their receptors, without new virions even accessing the cell. Hence, the productive infection of bacteriophages is stopped, generating no more viruses to invade the rest of the pathogenic population^25^.

The classification of bacteriophage depends on its nucleic acid type and its morphology. A Bacteriophage is composed of a head and a tail. The head or capsid is a protein shell that envelops the genetic material in an icosahedron shape. The tails generally have six tail fibers that vary in size and hold protein receptors to recognize the attachment sites on the surface of bacterial cell walls for attachment to specific host cells^26,27^. Tailed bacteriophages (*Caudovirales*) are divided into four families based on their tail shape (ICTV). *Myoviridae* has a long, rigid, and contractile tail with 80–485 nm length, and the average head diameter is 85 nm. *Siphoviridae* has a non-contractile long and flexible tail with a 79–535 nm length and an average head diameter of 55 nm. *Podoviridae* has a non-contractile short tail below 40 nm length and a 58 nm average head diameter^28^. Also, the newly created *Ackermannviridae* has up to four tail spike proteins and can infect a wide range of Gram-Negative bacteria^29^. In this study, isolated bacteriophage BC-S1 and ETEC-S3 morphology were analyzed using TEM. Based on the result, both phages showed an icosahedral head that is attached to a contractile tail. It can be assumed that both phages belong to the member of the *Caudovirales* order. Molecular analysis of the genome of the bacteriophage is required for further research to know the classification specifically and to ensure these phages do not contain any virulence-associated genes as well as antibiotic resistance genes^30^.

Since bacteriophages cause bacterial death, their potential use as food preservatives has become increasingly appealing. In this research, bacteriophage BC-S1 and BS-S2 were applied to cooked rice and pasteurized milk to control *B. cereus* and *B. subtilis* growth, and bacteriophage ETEC-S3 and EHEC-S4 were applied to chicken meat and fresh lettuce to control ETEC and EHEC growth. All isolated phages could significantly reduce their host bacteria population in every sample at all storage temperatures. Room temperature and low-temperature storage were selected because it was generally common temperature used to store food and beverage for a short period.

Overall, bacterial reduction in pasteurized milk samples was higher than in cooked rice, and bacterial reduction in chicken meat samples was higher than in lettuce. These reduction capabilities may be affected by the food sample matrices. Limited diffusion and contact of bacteria and phage were responsible for the low efficacy. Phage particles may be required to reduce bacterial contamination on moist food surfaces and in liquids compared to a drier food matrix because of the increased “mobility” of phages in the presence of moisture. The initial contact of bacteriophage and bacterium often occurs by diffusion and Brownian motion. Therefore, liquid samples such as pasteurized milk allowed greater diffusion of the phages rather than on the solid matrix such as cooked rice due to the restricted motions of the solvent molecule^28,31^.

Treatment for chicken meat showed a higher reduction than lettuce due to its natural juices. When chicken meat does not have space around it, the heat and moisture cannot escape, leaving the chicken to steam in its own juices. Phages in lettuce samples are likely unable to reach and invade bacteria, considering lettuce has a dry food matrix. The passive movement of phages across food surfaces is limited due to the lack of moisture^32^. On the other hand, at the same time and temperature, bacterial reduction in lettuce was higher compared with chicken meat in treatment using phage EHEC-S4. The situation is different on solid food with even surfaces like lettuce, where the total surface area and its ability to absorb liquid from the phage suspension are easier than uneven surface areas like chicken meat. Food with uneven and large surface area is difficult to treat with phage because phage distribution is physically limited to reach all bacterial targets. In addition, target bacteria may be embedded within the rather complex food matrix, shielding them from diffusing phage particles^33^.

Likewise, incubation at 28 °C showed higher bacterial reduction than at 4 °C of incubation. Temperature can also affect phage activity, where phages depend on the growth of bacterial hosts for their replication. *B. cereus*, *B. subtilis*, and *E. coli* grow in the mesophilic temperature range, with an optimum at about 37 °C. An optimal growth temperature of the bacterial host promotes a better replication of phage particles. In contrast, at a lower temperature, the rate of phage replication was considerably decreased or halted due to the lower growth rate of their hosts^34^.

All isolated bacteriophages in this study effectively reduced targeted food spoilage and foodborne pathogenic bacteria. However, further studies were required to determine their activities against other pathogens and their stability to various environmental conditions and characterize their genomic properties to ensure their safety if they want to be used as a preservation alternative.

## Methods

Bacteriophages were isolated from soil, then enriched and purified. Characterization of the isolated bacteriophages, including titer, host range, the efficiency of plating (EOP), the minimum inhibitory multiplicity of infection (miMOI), and morphology were determined. The application of the isolated bacteriophages to food samples was also evaluated.

### Inoculum preparation

*B. cereus* ATCC 10876, *B. subtilis* ATCC 6633, ETEC US Namru-1 and EHEC US Namru-1 were used in this study to serve as host strains for bacteriophage isolation. All bacterial strains were stored in 1.0 mL of aliquots of 20% (v/v) glycerol at − 80 °C. The bacterial cultures were inoculated onto Luria Bertani (LB) agar plates (Oxoid™) and were incubated at 37 °C overnight. The plates were kept at 4 °C and used as working cultures^35^.

### Sample collection

Soil near organic waste disposal was used as samples which were collected from Pakulonan Barat Village, Kelapa Dua Sub-district, Tangerang District of Banten Province, Indonesia. The soil samples were transported to the laboratory and processed for bacteriophage isolation.

### Bacteriophage isolation

Each bacterial host strain was grown in Luria Bertani (LB) broth (Oxoid™) to mid-log phase (OD_600_ = 0.132) by incubation at 37 °C, 120 rpm, overnight. Six grams of soil sample and 300 μL of each bacteria culture were added into 30 mL of LB broth. The samples were supplemented with 100 μL of 10 mM of CaCl_2_ and 100 μL of 0.5 mM of MgSO_4_ to enhance the bacteriophage growth^12^ and were incubated at 37 °C, 150 rpm, overnight. The samples were then centrifuged at 7000 × g for 15 min. The supernatant was filtered using a 0.22 μm pore-size disposable syringe filter (HIMEDIA) to remove the remaining bacterial cells. The filtrate was centrifuged again at 7000 × g for 10 min and tested for the presence of bacteriophages using the agar overlay assay^6,35^. Agar overlay assay was done by pouring top agar (LB consist of 0.6% agar) to the bottom agar (LB consist of 2% agar), where 150 μL of bacteriophage filtrate, 150 μL of mid-log phase bacterial host culture, 50 µL of 10 mM CaCl_2_, and 50 µL of 0.5 mM MgSO_4_ were mixed in 4 mL of molten LB soft agar and poured onto LB agar plate, then followed by incubation at 37 °C overnight. Clear plaque formation was observed^36^.

### Bacteriophage purification and enrichment

The isolated lytic bacteriophages were purified by stabbing the clear plaque gently using a sterile tip. The tip was then placed into 10 mL of LB Broth and pipetted up and down to release the bacteriophage particles. Bacteriophages were enriched by adding 250 µL of mid-log phase bacterial host culture into the LB Broth and incubated at 37 °C overnight at 120 rpm. After enrichment, the mixture was centrifuged at 7000 × g for 15 min and the supernatant was filtered using a 0.22 µm pore-size membrane filter (HIMEDIA) to obtain bacteriophage stock. The filtrates were kept in Ringer Solution (2.25 g NaCl, 0.105 g KCl, 0.12 g CaCl_2_, 0.05 g NaHCO_3_ in 500 mL distilled water) (Oxoid™) with a 1:1 ration (v/v) at 4 °C as a working solution for further analysis^37–39^.

### Bacteriophage titer determination

Titer were determined using the agar overlay assay method^37^. A series of tenfold dilutions of bacteriophage lysate solution were made using SM buffer (50 mM Tris-hydrochloride (Tris–HCl) [pH 7.5], 0.1 M NaCl, 8 mM magnesium sulphate heptahydrate (MgSO_4_•7H_2_O) and 0.01% (w/v) gelatine). Each dilution was plated according to agar overlay assay and incubated at 37 °C overnight. The number of visible plaques were calculated between 30 and 300 plaques which expressed as plaque forming unit per milliliter (PFU/mL)^35^.

### Host range determination

Isolated bacteriophages host range was determined using different species of the host bacteria, namely against *B*. *cereus* ATCC 10876, *B*. *subtilis* ATCC 6633, ETEC US Namru-1, EHEC US Namru-2, EPEC from US-Namru 2, and *V. cholerae* ATCC 14033. The isolated bacteriophage was tested against the different hosts to test their susceptibility with the agar overlay assay and incubated at 37 °C overnight^40^.

### Efficiency of plating (EOP)

EOP was tested using agar overlay assay and performed 3 times of replication and calculated by dividing the average PFU on target bacteria by the average PFU on host bacteria^21^.

### Minimum inhibitory multiplicity of infection (miMOI)

Bacterial host cultures were grown to mid-log phase and suspended to match 0.132 McFarland standard. The host culture and bacteriophage lysate were diluted to contain different MOI from 0.00001 to 100. Each of them was distributed 100 μL into the 96-well microtiter plate, then incubated at 37 °C for 10 h. The concentrations were determined every 1 h using microplate reader (Tecan Infinite® M200 PRO)^41^.

### Morphology analysis

Morphology of the isolated bacteriophages were determined using Transmission Electron Microscopy (TEM) at the Eijkman Institute for Molecular Biology, Jakarta, Indonesia. About 10 μL of bacteriophage was dropped on grid (400 mesh) and left for 30 s. Bacteriophage samples were negatively stained using 5 μL of 2% (w/v) uranyl acetate on carbon-coated grids. The grids were observed using JEM-1010 TEM (JEOL, Tokyo, Japan) at magnification of × 30,000^42,43^.

### Bacteriophage application on food samples

Cooked rice, pasteurized milk, chicken meat, and fresh lettuce were used as food samples. Raw chicken meat was cut into pieces (1 cm × 1 cm). Cooked rice and raw chicken meat were placed in 50 mL of Falcon tubes (Corning®) for approximately 1 g for each tube, whereas pasteurized milk (1 mL) was placed in 15 mL of Falcon tubes (Corning®). These samples were sterilized by autoclaving for 15 min at 121 °C to kill all natural bacteria^33^. Meanwhile, fresh lettuces were rinsed with clean water, followed by swabbed with 96% of alcohol on its surfaces. The lettuces were cut into pieces (1 cm × 1 cm) and placed into 50 mL Falcon tubes (Corning®). Then the tubes were exposed to UV light from laminar airflow (ESCO) for about 45 min^44^.

After sterilization, each sample of cooked rice and pasteurized milk were inoculated with 100 μL of mid-log phase bacterial host strain suspensions (*B. cereus* and *B. subtilis*) and 100 μL of isolated bacteriophages (BC-S1 and BS-S2) which were diluted to contain MOI of 0.1. While each sample of chicken meat and fresh lettuce were inoculated with 100 μL of mid-log phase bacterial host strain suspensions (ETEC and EHEC) and 100 μL of isolated bacteriophages (ETEC-S3 and EHEC-S4) which were diluted to contain MOI 0.001 for ETEC and MOI 1 for EHEC. All samples were then incubated at 4 °C and 28 °C overnight^45^.

After the incubation, 10 mL of SM buffer was added to each sample and the tubes were vortexed for around 3 min. Each sample was then serially diluted and spread onto LB agar plate, incubated at 37 °C overnight. For positive control, food samples were inoculated with host strain only. For negative control, food samples were inoculated with isolated bacteriophage lysate only. Colonies were counted between 30 and 300 colonies which expressed as colony forming unit per milliliter (CFU/mL)^46^.

### Statistical analysis

The data were collected after 3 times of replication and statistical analysis was done using one-way ANOVA followed by Tukey’s-B test (SPSS Inc. IBM corporation). The level of difference was defined at P ≤ 0.05, and different letters in each column indicated significant differences from other samples. For control-treatment pairing of each sample, its significant reduction was determined using the paired-samples T-Test with the level of differences defined at P ≤ 0.05^47^.

## Conclusions

Four lytic bacteriophages, BC-S1, BS-S2, ETEC-S3, and EHEC-S4 were successfully recovered from soil samples. They were considered highly specific phages with a narrow spectrum of host range, where phage ETEC-S3 was found inefficient against B. cereus, and phage EHEC-S4 had low efficiency against EPEC. This narrow property is desirable because it usually has great specificity to the host. By using TEM, phage BC-S1, and BS-S2 could be categorized as one of the Caudovirales members. These phages showed a significant reduction in food samples on miMOI of 0.1 for both phage BC-S1 and BS-S2, miMOI 0.001 for phage ETEC-S3, and miMOI 1 for phage EHEC-S4 at 4 °C and 28 °C storage temperature. These results showed the potential efficacy of bacteriophage in reducing targeted food spoilage and foodborne bacteria. Thus, it is also promising to be studied further.

### Limitation

This study only screened some of the food spoilage and foodborne pathogenic bacteria, also the food that has been assayed is limited, therefore other microbes and food samples need to be explored. On the other hand, it is also should be characterized further for their genomic properties like virulence factor and antibiotic resistance genes also the survival of this bacteriophage in various food processing conditions.

## Data Availability

The data of this study is available with the corresponding author upon request.

## Abbreviations

EHEC: Enterohemorrhagic *Escherichia coli*
EOP: Efficiency of Plating
EPEC: Enteropathogenic *Escherichia coli*
ETEC: Enterotoxigenic *Escherichia coli*
miMOI: Minimum Inhibitory Multiplicity of Infection
SM: Sodium-Magnesium buffer
TEM: Transmission Electron Microscopy

## References

[1] Nerin C, Aznar M, Carrizo D (2016). Food contamination during food process. Trends Food Sci. Technol..

[2] Rawat S (2015). Food spoilage: Microorganisms and their prevention. Asian J. Plant Sci. Res..

[3] Viedma PM, Abriouel H, Omar NB, Lopez RL, Galves A (2011). Inhibition of spoilage and toxigenic *Bacillus* species in dough from wheat flour by the cyclic peptide enterocin AS-48. Food Control.

[4] Moschonas G, Lianou A, Nychas GE, Panagou EZ (2021). Spoilage potential of *Bacillus subtilis* in a neutral-pH dairy dessert. Food Microbiol..

[5] Yang SC, Lin CH, Aljuffali IA, Fang JY (2017). Current pathogenic *Escherichia coli* foodborne outbreak cases and therapy development. Arch. Microbiol..

[6] Oh H (2017). Isolation and characterization of *Bacillus cereus* bacteriophages from foods and soil. Food Environ. Virol..

[7] Dwivedi S, Prajapati P, Vyas N, Malviya S, Kharia A (2017). A review on food preservation: Methods, harmful effects and better alternatives. Asian J. Pharm. Pharmacol..

[8] Sulakvelidze A (2013). Using lytic bacteriophages to eliminate or significantly reduce contamination of food by foodborne bacterial pathogens. J. Sci. Food Agric..

[9] Wang J, Kanach A, Han R, Applegate B (2021). Application of bacteriophage in rapid detection of *Escherichia coli* in foods. Curr. Opin. Food Sci..

[10] Jamal M (2018). Bacteriophages: An overview of the control strategies against multiple bacterial infections in different fields. J. Basic Microbiol..

[11] Shende RK, Hirpukar SD, Sannat C, Rawat N, Pandey V (2017). Isolation and characterization of bacteriophages with lytic activity against common bacterial pathogens. Vet. World.

[12] Vikram A, Woolston J, Sulakvelidze A (2021). Phage biocontrol applications in food production and processing. Curr. Issues Mol. Biol..

[13] Anderson B (2011). Enumeration of bacteriophage particles: Comparative analysis of the traditional plaque assay and real time QPCR- and nanosight-based assays. Bacteriophage.

[14] Hyman P (2019). Phages for phage therapy: Isolation, characterization, and host range breadth. Pharmaceuticals.

[15] Doss J, Culbertson K, Hahn D, Camacho J, Barekzi N (2017). A review of phage therapy against bacterial pathogens of aquatic and terrestrial organisms. Viruses.

[16] Sritha KS, Bhat SG (2018). Genomics of *Salmonella* phage ΦStp1: Candidate bacteriophage for biocontrol. Virus Genes.

[17] Jończyk-Matysiak E (2019). Factors determining phage stability/activity: Challenges in practical phage application. Expert Rev. Anti-infect. Ther..

[18] Ly-Chatain MH (2014). The factors affecting effectiveness of treatment in phages therapy. Front. Microbiol..

[19] Jonge PA, Nobrega FL, Brouns SJJ, Dutilh BE (2018). Molecular and evolutionary determinants of bacteriophage host range. Trends Microbiol..

[20] Kan S, Fornelos N, Schuch R, Fischetti VA (2013). Identification of a ligand on the wip1 bacteriophage highly specific for a receptor on *Bacillus anthracis*. J. Bacteriol..

[21] Mirzaei MK, Nilsson AS (2015). Isolation of phages for phage therapy: A comparison of spot test and efficiency of plating analyses for determination of host range and efficacy. PLoS ONE.

[22] Azeredo J, Sillankorva S (2018). Bacteriophage Therapy: From Lab to Clinical Practice.

[23] Abedon ST (2016). Phage therapy dosing: The problem(s) with multiplicity of infection (MOI). Bacteriophage.

[24] Ssekatawa K (2021). A review of phage mediated antibacterial applications. Alexandr. J. Med..

[25] Abd-Allah IM, Housseiny GSE, Yahia IS, Aboshanab KM, Hassouna NA (2021). Rekindling of a masterful precedent; bacteriophage: Reappraisal and future pursuits. Front. Cell. Infect. Microbiol..

[26] Mansour NM (2017). Bacteriophages are natural gift, could we pay further attention. J. Food Microbiol..

[27] Gourkhede DP (2020). Application of bacteriophages in food industry: A review. Int. J. Livest. Res..

[28] Harada LK (2018). Biotechnological applications of bacteriophages: State of art. Microbiol. Res..

[29] Sørensen AN, Woudstra C, Sørensen MCH, Brøndsted L (2021). Subtypes of tail spike proteins predicts the host range of *Ackermannviridae* phages. Comput. Struct. Biotechnol. J..

[30] Fokine A, Rossmann MG (2014). Molecular architecture of tailed double-stranded DNA phages. Landes Biosci..

[31] Lewis R, Hill C (2020). Overcoming barriers to phage application in food and fed. Curr. Opin. Biotechnol..

[32] Moye ZD, Woolston J, Sulakvelidze A (2018). Bacteriophage applications for food production and processing. Viruses.

[33] Guenther S, Huwyler D, Richard S, Loessner MJ (2009). Virulent bacteriophage for efficient biocontrol of *Listeria monocytogenes* in ready-to-eat foods. Food Microbiol..

[34] Duc HM, Son HM, Honjoh KI, Miyamoto T (2018). Isolation and application of bacteriophages to reduce Salmonella contamination in raw chicken meat. LWT Food Sci. Technol..

[35] Thung TY (2017). Isolation of food-borne pathogen bacteriophages from retail food and environmental sewage. Food. Res..

[36] Adams MH (1959). Bacteriophages.

[37] Salifu SP, Casey SAC, Foley S (2013). Isolation and characterization of soilborne virulent bacteriophages infecting the pathogen *Rhodococcus equi*. J. Appl. Microbiol..

[38] Gencay YE, Birk T, Sørensen MCH, Brøndsted L (2016). Methods for isolation, purification, and propagation of bacteriophages of *Campylobacter jejuni*. Methods Mol. Biol..

[39] Arivo D, Rusmana I, Budiarti S (2016). Isolation and characterization of EPEC from domestic waste in Indonesia. Malay. J. Pathol..

[40] Budiarti S, Pratiwi RH, Rusmana I (2011). Infectivity of lytic phage to enteropathogenic *Escherichia coli* from diarrheal patients in Indonesia. J US-China Med. Sci..

[41] Vipra A (2013). Determining the minimum inhibitory concentration of bacteriophages: Potential advantages. J. Adv. Microbiol..

[42] Litt PK, Jaroni D (2017). Isolation and physiomorphological characterization of *Escherichia coli* O157:H7-infecting bacteriophages recovered from beef cattle operations. Int. J. Microbiol..

[43] Lee WJ, Billington C, Hudson JA, Heinemann JA (2011). Isolation and characterization of phages infecting *Bacillus cereus*. Lett. Appl. Microbiol..

[44] El-Shibiny A, El-Sahhar S, Adel M (2017). Phage applications for improving food safety and infection control in Egypt. J. Appl. Microbiol..

[45] Shin H, Bandara N, Shin E, Ryu S, Kim K (2011). Prevalence of *Bacillus cereus* bacteriophages in fermented foods and characterization of phage JBP901. Res. Microbiol..

[46] Hudson JA, Billington C, Wilson T, On SLW (2013). Effect of phage and host concentration on the inactivation of *Escherichia coli* O157:H7 on cooked and raw beef. Food Sci. Technol. Int..

[47] Lukman C, Yonathan C, Magdalena S, Waturangi DE (2020). Isolation and characterization of pathogenic *Escherichia coli* bacteriophages from chicken and beef offal. BMC Res. Notes.

